# Opioid use and subsequent delirium risk in patients with advanced cancer in palliative care: a multicenter registry study

**DOI:** 10.1038/s41598-024-56675-1

**Published:** 2024-03-12

**Authors:** Shin Hye Yoo, Jiseung Kang, Hyeon Jin Kim, Si Won Lee, Moonki Hong, Eun Hee Jung, Yu Jung Kim, Dong Keon Yon, Beodeul Kang

**Affiliations:** 1 Center for Palliative Care and Clinical Ethics, Seoul National University Hospital, Seoul, South Korea; 2 Department of Anesthesia, Critical Care and Pain Medicine, Massachusetts General Hospital, Boston, MA USA; 3 Division of Sleep Medicine, Harvard Medical School, Boston, MA USA; 4 Center for Digital Health, Medical Science Research Institute, Kyung Hee University College of Medicine, Seoul, South Korea; 5 Department of Regulatory Science, Kyung Hee University, Seoul, South Korea; 6 Division of Medical Oncology, Department of Internal Medicine, Yonsei Cancer Center, Yonsei University Health System, Seoul, South Korea; 7 Palliative Cancer Center, Yonsei Cancer Center, Yonsei University Health System, Seoul, South Korea; 8 Division of Hematology and Medical Oncology, Department of Internal Medicine, Seoul National University Bundang Hospital, Seoul National University College of Medicine, Seongnam, South Korea; 9 Department of Pediatrics, Kyung Hee University Medical Center, Kyung Hee University College of Medicine, 23 Kyungheedae-Ro, Dongdaemun-Gu, Seoul, 02447 South Korea; 10 Division of Medical Oncology, Department of Internal Medicine, CHA Bundang Medical Center, CHA University School of Medicine, 59 Yatap-Ro, Bundang-Gu, Seongnam, 13496 South Korea

**Keywords:** Opioid, Delirium, Cancer, Palliative care, Oncology, Risk factors

## Abstract

The prevalent use of opioids for pain management in patients with advanced cancer underscores the need for research on their neuropsychiatric impacts, particularly delirium. Therefore, we aimed to investigate the potential association between opioid use and the risk of delirium in patients with advanced cancer admitted to the acute palliative care unit. We conducted a retrospective observational study utilizing a multicenter, patient-based registry cohort by collecting the data from January 1, 2019, to December 31, 2020, in South Korea. All data regarding exposures, outcomes, and covariates were obtained through retrospective chart reviews by a team of specialized medical professionals with expertise in oncology. Full unmatched and 1:1 propensity-score matched cohorts were formed, and stratification analysis was conducted. The primary outcome, delirium, was defined and diagnosed by the DSM-IV. Of the 2,066 patients with advanced cancer, we identified 42.8% (mean [SD] age, 64.4 [13.3] years; 60.8% male) non-opioid users and 57.2% (62.8 [12.5] years; 55.9% male) opioid users, respectively. Opioid use was significantly associated with an increased occurrence of delirium in patients with advanced cancer (OR, 2.02 [95% CI 1.22–3.35]). The risk of delirium in patients with advanced cancer showed increasing trends in a dose-dependent manner. High-dose opioid users showed an increased risk of delirium in patients with advanced cancer compared to non-opioid users (low-dose user: OR, 2.21 [95% CI 1.27–3.84]; high-dose user: OR, 5.75 [95% CI 2.81–11.77]; ratio of OR, 2.60 [95% CI 1.05–6.44]). Patients with old age, male sex, absence of chemotherapy during hospitalization, and non-obese status were more susceptible to increased risk of delirium in patients with cancer. In this multicenter patient-based registry cohort study, we found a significant, dose-dependent association between opioid use and increased risk of delirium in patients with advanced cancer. We also identified specific patient groups more susceptible to delirium. These findings highlight the importance of opioid prescription in these patients with advanced cancer, balancing effective doses for pain management and adverse dose-inducing delirium.

## Introduction

Delirium, a common and acute neuropsychiatric complication in patients with advanced cancer^1,2^, remains significantly underrecognized and undetermined among patients with advanced cancer^3^. Characterized by a fluctuating disturbance in attention and awareness^4^, delirium adversely impacts the disease course by impairing communication and hindering the participation of patients in care, such as treatment decisions, counseling, and diagnosis^5^. Although frequently reported in advanced cancer, previous researches on delirium are predominantly limited to case reports and small patients cohorts^6–8^.

The etiology of delirium in these patients is typically multifactorial, and our prior research identified several risk factors for delirium in patients with cancer, including old age, absence of chemotherapy during hospitalization, hearing impairment, underweight status, current opioid use, and history of delirium and other psychiatric disorders^9^. Opioid prescriptions, in particular, have been identified in various studies as potential triggers of delirium, observed across different surgical and disease groups, including those with neurological injury, pain, infection, fever, and hypotension^4,10,11^. However, previous studies exploring the association between opioid use and delirium in patients with advanced cancer showed controversy and limited cohort size^12–15^.

The inevitability of using analgesics, particularly opioids, in managing chronic pain in patients with cancer, especially those with advanced stage, further complicates this issue^16^. In cancer pain management, approximately 50% of patients are estimated to use analgesics, with opioids being the choice for half of these individuals^17^. This significant reliance on opioids, despite their critical role in pain management, raises concerns about their neuropsychiatric side effects, particularly delirium, in patients with advanced cancer. Addressing this concern, we aimed to investigate the association between opioid use and the occurrence of delirium in patients with advanced cancer admitted to the acute palliative care unit (APCU), by utilizing a large-scale, multicenter, patient-based registry cohort.

## Methods

### Data source

In this retrospective observational study, we constructed a patient-based multicenter registry cohort by collecting data from four centers, including Seoul National University Hospital, Seoul National University Bundang Hospital, Yonsei University Severance Hospital, and CHA University Bundang Medical Center in South Korea. This patient-based registry cohort is distinguished by the following strengths^9^: (1) Four academic and accredited cancer centers compilated the data; (2) A team of specialized medical professionals with expertise in oncology was responsible for constructing and managing the dataset; (3) Patients who received supportive care during treatment and those who terminated their treatment were incorporated into the study; and (4) Since all of the analyzed data were anonymized, the requirement for prior consent was unnecessary. The Institutional Review Boards of the four centers (Seoul National University, H-2103-028-1201; Seoul National University Bundang Hospital, B-2104/681-405; Yonsei University, 4-2021-0323; and CHA University, CHAMC 2021-03-054-002) approved the protocol. The requirement for informed consent was waived by the four centers (Seoul National University; Seoul National University Bundang Hospital; Yonsei University; and CHA University) because only retrospective anonymized data were examined. This research adhered to the ethical guidelines established by relevant national and institutional review boards for human research and followed the 1975 Helsinki Declaration, as amended in 2008.

### Study design and population

This study incorporated 2152 patients with advanced cancer admitted to the ACPU of four centers from January 1, 2019, to December 31, 2020, with follow-up until the date of death or March 31, 2022, based on the admission date. Exclusions were made based on: (1) hospital stay exceeding three months (excluded, n = 5); (2) transfers to other departments (excluded, n = 6); (3) observation of terminal delirium (excluded, n = 3); (4) patient with a history of delirium (excluded, n = 55); and (5) missing baseline characteristics of study subjects (excluded, n = 17). If delirium occurs within two weeks before death, we define it as terminal delirium. Following these criteria, 2066 individuals were included in the analysis. The assessment of opioid use, delirium, and other covariates was conducted through meticulous retrospective chart reviews, ensuring a thorough evaluation of patient histories and clinical outcomes.

### Exposure

Opioid exposure was considered for patients who received opioid medications during hospitalization. The morphine equivalent daily dose (MEDD) was used to assess exposed dose levels. We used a cutoff of the upper 25% of the MEDD (100 mg MEDD) threshold to categorize low-dose and high-dose users. Prescriptions of medications were conducted through medical specialists.

### Outcome

The primary outcome, delirium in patients with advanced cancer, was identified via medical records and diagnosed based on the Diagnostic and Statistical Manual of Mental Disorders, Fifth Edition (DSM-5)^18^. At least two medical specialists performed a detailed review and recorded potentially related symptoms and associated medications. In cases of conflicting opinions, additional experts participated in the diagnosis and voted to reach a conclusion^9^.

### Covariates

The study considered the following various patient related covariates: age (< 50, 50–59, 60–69, and ≥ 70 years), sex, the status of chemotherapy during hospitalization, living with family, eligibility for medical aid, education level (high school graduated or under, college graduated or higher, and unknown), visual impairment (wearing glasses), hearing impairment (using hearing aids), alcohol consumption (non-drinker, 1–3 times a week, and ≥ 4 times a week), smoking (non-smoker, ex-smoker, and current smoker), obesity (< 18.5 kg/m^2^ [underweight], 18.5–25 kg/m^2^ [normal], 25–30 kg/m^2^ [overweight], and ≥ 30 kg/m^2^ [obese])^19–21^, blood pressure (systolic blood pressure [SBP] ≥ 140 mmHg or diastolic blood pressure [DBP] ≥ 90 mmHg and SBP < 140 mmHg and DBP < 90 mmHg)^22^, body temperature (normal [< 38 ℃] and hyperthermia [≥ 38 ℃]), operation received, current cancer treatment status (cytotoxic chemotherapy, immunotherapy, targeted chemotherapy, radiation therapy, no further treatment, and other), use of concomitant medication (sedatives, antidepressant, antiepileptic, cholinergic, and anticholinergic), and history of diseases, such as cardiovascular disease, diabetes mellitus, respiratory disease, liver, mental illness, and head injury. These all variables were obtained through medical chart reviews and categorized according to the International Classification of Diseases, 10th edition (ICD-10)^23,24^.

### Statistical analysis

**Figure 1:**
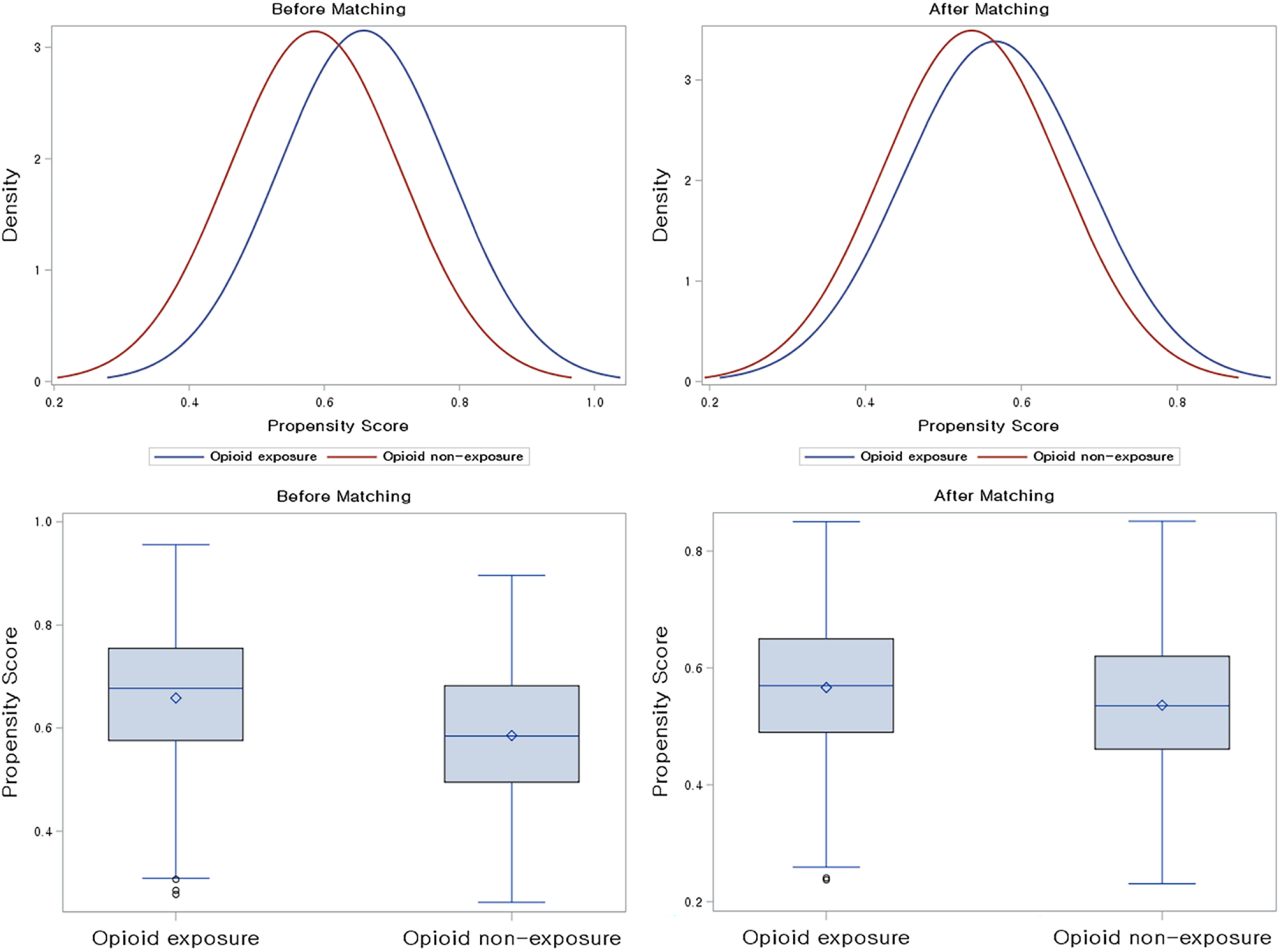
Density plot and box plot of 1:1 propensity score matching cohort.

In this study, we aimed to investigate the association between opioid use and the development of delirium in patients with advanced cancer. To control for potential confounding variables and balance demographic characteristics between comparison groups, we constructed a propensity score (PS)-matching cohort (Fig. 1)^21,25–27^. All variables listed in Table 1 were used for matching, with PS calculated through a multivariate logistic regression model. Individuals with PS differences within the specified caliper (0.1) were matched in a 1:1 ratio using the greedy nearest-neighbor algorithm. Finally, 776 patients were allocated to each of the opioid-exposed and unexposed groups. The adequacy of PS matching was evaluated by standardized mean differences (SMD), with an SMD less than 0.1 indicating no significant imbalance^25^.

**Table 1:** Baseline characteristics of study subjects.

	Full unmatched cohort (n = 2066)		1:1 PS-matched cohort (n = 1552)		
	Non-opioid user	Opioid user	Non-opioid user	Opioid user	SMD*
Total, n	884	1,182	776	776	
Age (years; mean, SD)	64.4 (13.3)	62.8 (12.5)	63.5 (13.4)	63.5 (12.5)	< 0.01
Age group (years; n, %)					0.05
< 50	111 (12.6)	168 (14.2)	109 (14.1)	99 (12.8)	
50–59	179 (20.3)	256 (21.7)	163 (21.0)	161 (20.8)	
60–69	255 (28.9)	399 (33.8)	239 (30.8)	253 (32.6)	
≥ 70	339 (38.4)	359 (30.4)	265 (34.2)	263 (33.9)	
Sex (n, %)					0.01
Male	537 (60.8)	661 (55.9)	456 (58.8)	454 (58.5)	
Female	347 (39.3)	521 (44.1)	320 (41.2)	322 (41.5)	
Chemotherapy during hospitalization (n, %)					0.02
Yes	309 (35.0)	273 (23.1)	254 (32.7)	246 (31.7)	
No	575 (65.1)	909 (76.9)	522 (67.3)	530 (68.3)	
Living with family (n, %)					0.05
Yes	547 (61.9)	833 (70.5)	490 (63.1)	507 (65.3)	
No	337 (38.1)	349 (29.5)	286 (36.9)	269 (34.7)	
Medical aid recipients (n, %)					0.01
Yes	33 (3.7)	61 (5.2)	27 (3.5)	26 (3.4)	
No	851 (96.3)	1,121 (94.8)	749 (96.5)	750 (96.7)	
Education level (n, %)					0.03
High school graduated or under	357 (40.4)	591 (50.0)	328 (42.3)	336 (43.3)	
College graduated or higher	199 (22.5)	316 (26.7)	194 (25.0)	188 (24.2)	
Unknown	328 (37.1)	275 (23.3)	254 (32.7)	252 (32.5)	
Visual impairment (wearing glasses; n, %)					0.01
Yes	51 (5.8)	53 (4.5)	44 (5.7)	43 (5.5)	
No	833 (94.2)	1,129 (95.5)	732 (94.3)	733 (94.5)	
Hearing impairment (using hearing aids; n, %)					0.03
Yes	12 (1.4)	9 (0.8)	6 (0.8)	8 (1.0)	
No	872 (98.6)	1,173 (99.2)	770 (99.2)	768 (99.0)	
Alcohol consumption (n, %)					< 0.01
Non-drinker	751 (85.0)	988 (83.6)	667 (86.0)	664 (85.6)	
1–3 times a week	82 (9.3)	140 (11.8)	72 (9.3)	69 (8.9)	
≥ 4 times a week	51 (5.8)	54 (4.6)	37 (4.8)	43 (5.5)	
Smoking (n, %)					0.05
Non-smoker	632 (71.5)	791 (66.9)	555 (71.5)	544 (70.1)	
Ex-smoker	27 (3.1)	22 (1.9)	19 (2.5)	18 (2.3)	
Current smoker	225 (25.5)	369 (31.2)	202 (26.0)	214 (27.6)	
Obesity (n, %)^†^					0.08
Underweight	172 (19.5)	274 (23.2)	160 (20.6)	165 (21.3)	
Normal weight	546 (61.8)	729 (61.7)	476 (61.3)	481 (62.0)	
Overweight	139 (15.7)	160 (13.5)	122 (15.7)	112 (14.4)	
Obese	27 (3.1)	19 (1.6)	18 (2.3)	18 (2.3)	
Blood pressure (n, %)					0.02
SBP ≥ 140 mmHg or DBP ≥ 90 mmHg	685 (77.5)	877 (74.2)	598 (77.1)	605 (78.0)	
SBP < 140 mmHg and DBP < 90 mmHg	199 (22.5)	305 (25.8)	178 (22.9)	171 (22.0)	
Body temperature (n, %)					0.01
Normal temperature (< 38 ℃)	847 (95.8)	1,131 (95.7)	741 (95.5)	742 (95.6)	
Hyperthermia (≥ 38 ℃)	37 (4.2)	51 (4.3)	35 (4.5)	34 (4.4)	
Operation (n, %)					0.01
Yes	484 (54.8)	695 (58.8)	435 (56.1)	430 (55.4)	
No	400 (45.3)	487 (41.2)	341 (43.9)	346 (44.6)	
Cancer treatment (n, %)					0.10
Cytotoxic chemotherapy	425 (48.1)	353 (29.9)	350 (45.1)	337 (43.4)	
Immunotherapy	66 (7.5)	84 (7.1)	63 (8.1)	61 (7.9)	
Targeted chemotherapy	76 (8.6)	64 (5.4)	61 (7.9)	58 (7.5)	
Radiation Therapy	41 (4.6)	47 (4.0)	37 (4.8)	37 (4.8)	
No further treatment	257 (29.1)	625 (52.9)	254 (32.7)	274 (35.3)	
Other	19 (2.2)	09 (0.8)	11 (1.4)	9 (1.2)	
Use of concomitant medication					
Sedatives (n, %)	111 (12.6)	230 (19.5)	102 (13.1)	123 (15.9)	0.08
Antidepressant (n, %)	33 (3.7)	91 (7.7)	31 (4.0)	35 (4.5)	0.03
Antiepileptic (n, %)	54 (6.1)	91 (7.7)	50 (6.4)	54 (7.0)	0.02
Cholinergic (n, %)	21 (2.4)	28 (2.4)	19 (2.5)	20 (2.6)	0.01
Anticholinergic (n, %)	16 (1.8)	18 (1.5)	12 (1.6)	16 (2.1)	0.04
History of disease					
Cardiovascular (n, %)	338 (38.2)	454 (38.4)	290 (37.4)	304 (39.2)	0.04
Diabetes mellitus (n, %)	184 (20.8)	268 (22.7)	161 (20.8)	172 (22.2)	0.03
Respiratory (n, %)	83 (9.4)	99 (8.4)	71 (9.2)	70 (9.0)	< 0.01
Liver (n, %)	57 (6.5)	76 (6.4)	52 (6.7)	48 (6.2)	0.02
Mental illness (n, %)	39 (4.4)	84 (7.1)	38 (4.9)	43 (5.5)	0.03
Head injury (n, %)	56 (6.3)	82 (6.9)	50 (6.4)	51 (6.6)	0.01

*DBP* diastolic blood pressure, *PS* propensity matching, *SBP* systolic blood pressure, *SD* standard deviation, *SMD* standardized mean difference.

^†^Obesity (body mass index, kg/m^2^) was categorized as < 18.5 kg/m^2^ (underweight), 18.5–25.0 kg/m^2^ (normal), 25.0–30.0 kg/m^2^ (overweight), and ≥ 30.0 kg/m^2^ (obese).

*SMD < 0.1 indicates no major imbalance.

Odds ratio (OR) with 95% confidence intervals (CIs) using binary logistic regression models were used for estimation^27^. In addition, an adjusted model was used to minimize the impact of potential confounders, incorporating the following variables: age, sex, chemotherapy during hospitalization, living with family, medical aid recipients, alcohol consumption, smoking, and obesity. Statistical significance was established at a two-sided P value < 0.05. All analyses and visualization were performed using SAS software (version 9.4; SAS Institute Inc., Cary, NC, USA) and R software (version 4.1.0; R Foundation for Statistical Computing, Vienna, Austria)^28,29^.

### Ethical approval

The Korean government anonymized all patient-related data, including personal identification numbers, to enhance confidentiality. The protocol was approved by the institutional review boards of the four centers (CHA University, CHAMC 2021-03-054-002; Seoul National University, H-2103-028-1201; Seoul National University Bundang Hospital, B-2104/681-405; and Yonsei University, 4-2021-0323). We conducted this study using de-identified administrative data that were obtained without prior consent.

## Results

Of the 2066 eligible patients with cancer admitted to the APCU, we identified 42.8% (884/2,066) non-opioid users (mean [standard deviation, SD] age, 64.4 [13.3] years; 60.8% male) and 57.2% (1182/2124) opioid users (62.8 [12.5] years; 55.9% male) in the full matched cohort (Table 1). Following the 1:1 PS matching, the SMD values were below 0.1, suggesting no major imbalances in the baseline characteristics. Table 1 details these baseline demographic characteristics.

**Figure 2:**
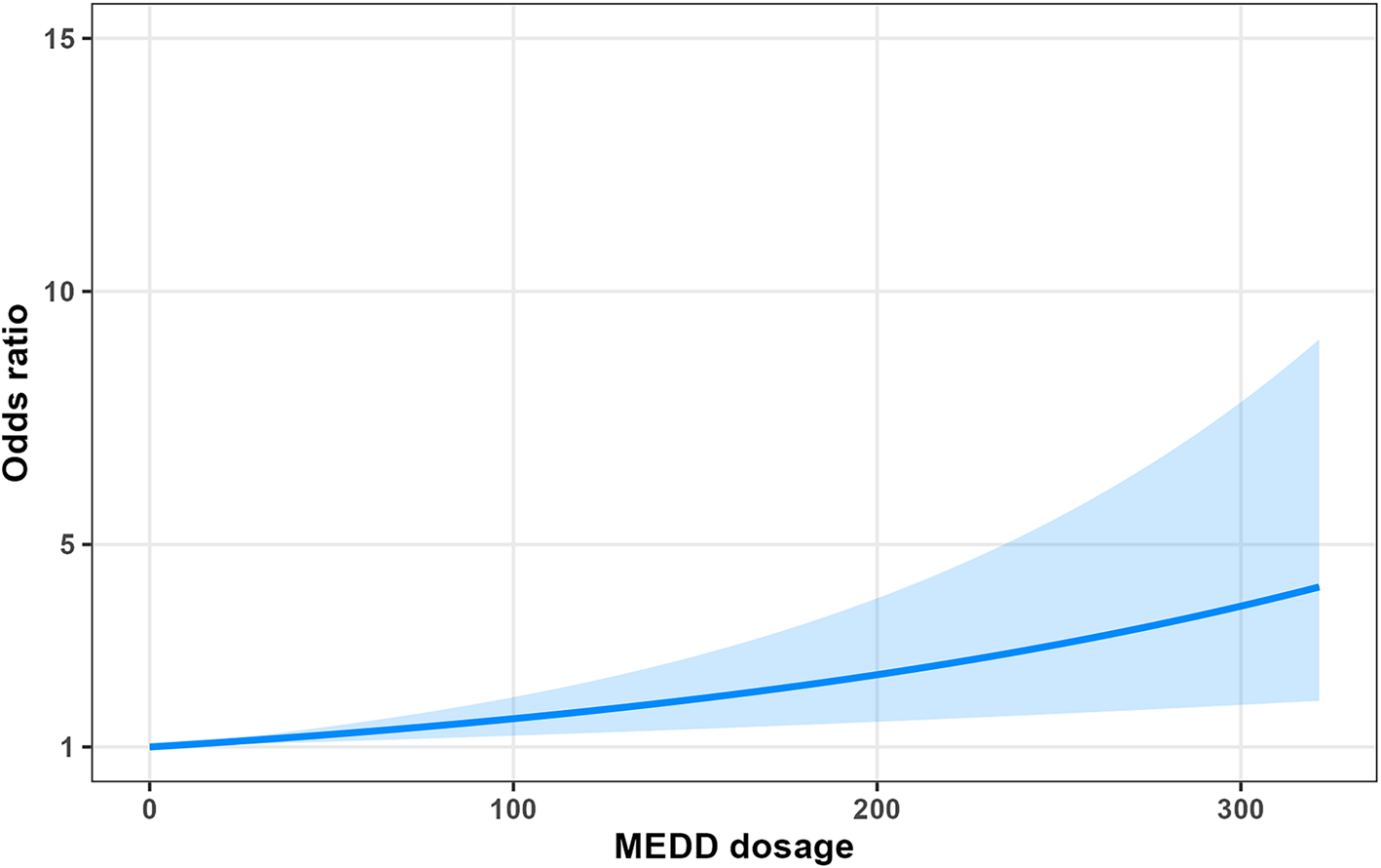
Dose-dependent association between MEDD and incidence of delirium. Morphine equivalent daily dose.

Opioid use was associated with an increased occurrence of delirium in patients with advanced cancer (OR, 2.02 [95% CI 1.22–3.35]) in Table 2. In particular, high-dose opioid users showed an increased risk of delirium in patients with advanced cancer compared to non-opioid users (low-dose user: OR, 2.21 [95% CI 1.27–3.84]; high-dose user: OR, 5.75 [95% CI 2.81–11.77]; ratio of OR, 2.60 [95% CI 1.05–6.44]). Furthermore, the risk of delirium in patients with advanced cancer showed increasing trends in a dose-dependent manner (Table 2 and Fig. 2).

**Table 2:** Odds ratio models for the association between opioid use and delirium in patients with advanced cancer with the 1:1 propensity-score-matched cohort.

	Delirium event/exposed, n (%)	Crude OR (95% CI)	Adjusted OR (95% CI)^†^
Opioid use			
Non-user	25/752 (3.32)	1.00 (reference)	1.00 (reference)
User	46/752 (6.12)	**1.90 (1.15–3.12)**	**2.02 (1.22–3.35)**
Dose-dependent association (MEDD)			
Non	22/776 (2.84)	1.00 (reference)	1.00 (reference)
Low-dose user	36/647 (5.56)	**2.02 (1.18–3.47)**	**2.21 (1.27–3.84)**
High-dose user	15/129 (11.63)	**4.51 (2.27–8.95)**	**5.75 (2.81–11.77)**
Ratio of OR (High-dose vs. Low-dose [ref])	–	2.23 (0.93–5.35)	**2.60 (1.05–6.44)**

CI, confidence interval; MEDD, morphine equivalent daily dose; OR, odds ratio.

^†^The model was adjusted for age (< 50, 50–59, 60–69, and ≥ 70 years), sex, chemotherapy during hospitalization, living with family, medical aid recipients, alcohol consumption (0, 1–3, and ≥ 4), smoking (0, 1–3, and ≥ 4), and body mass index (< 18.5, 18.5–25.0, 25.0–30.0, ≥ 30.0 kg/m^2^).

Numbers in bold indicate significant differences (p < 0.05).

**Figure 3:**
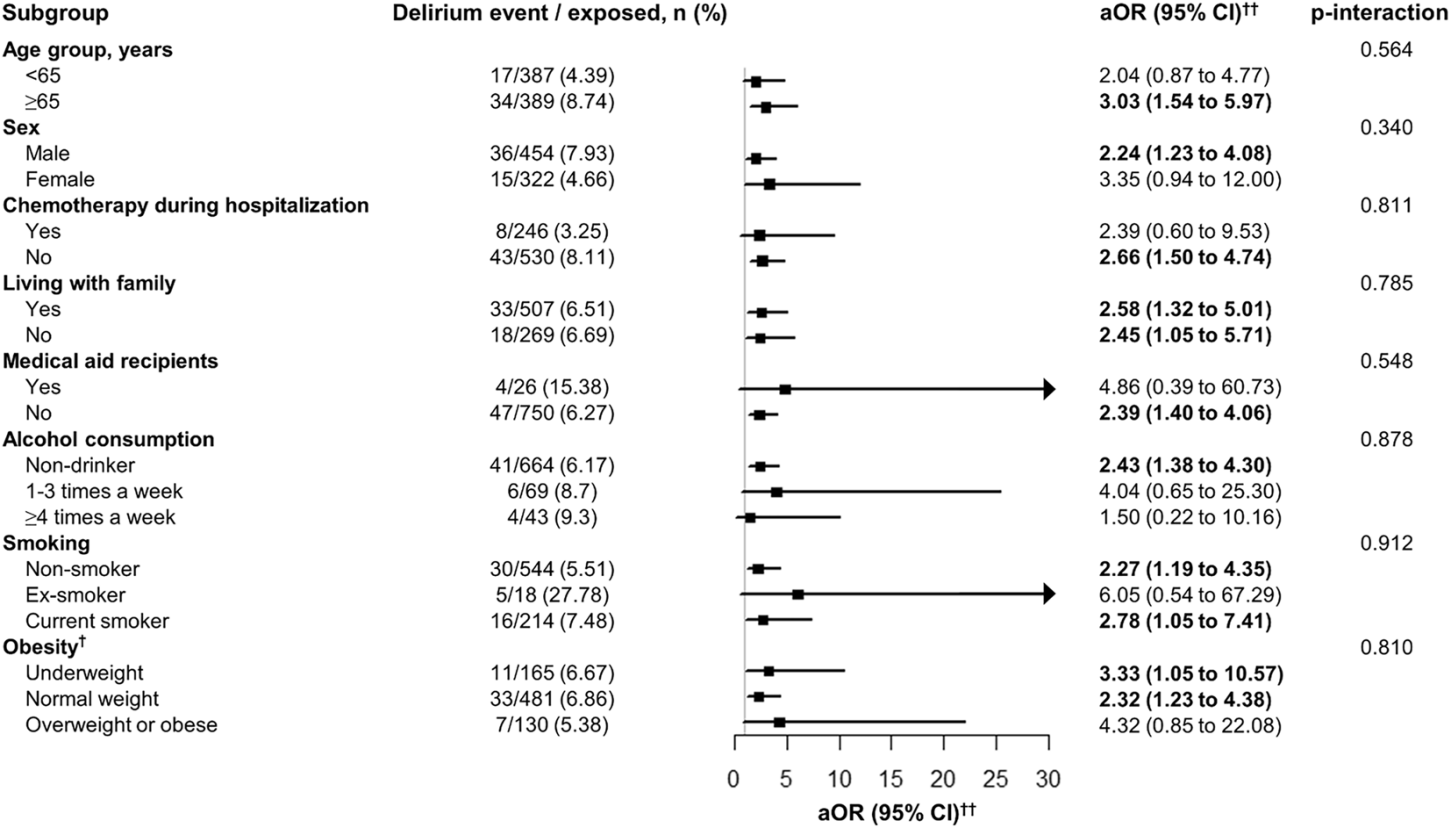
Stratification analysis for odds ratio models of the association between opioid use and delirium in patients with advanced cancer with the 1:1 propensity-score-matched cohort. *CI* confidence interval, *MEDD* morphine equivalent daily dose, *aOR* adjusted odds ratio. ^†^Obesity (body mass index, kg/m^2^) was categorized as < 18.5 kg/m^2^ (underweight), 18.5–25.0 kg/m^2^ (normal), 25.0–30.0 kg/m^2^ (overweight), and ≥ 30 kg/m^2^ (obese). ^††^The model was adjusted for age (< 50, 50–59, 60–69, and ≥ 70 years), sex, chemotherapy during hospitalization, living with family, medical aid recipients, alcohol consumption (0, 1–3, and ≥ 4), smoking (0, 1–3, and ≥ 4), and body mass index (< 18.5, 18.5–25.0, 25.0–30.0, ≥ 30.0 kg/m^2^). Numbers in bold indicate significant differences (p < 0.05).

Stratification analysis was performed in 1:1 PS-matched cohorts (Fig. 3). Higher occurrences of delirium in patients with advanced cancer showed in individuals with old age (OR, 3.03 [95% CI 1.54–5.97]), male sex (2.24 [95% CI 1.23–4.08]), absence of chemotherapy during hospitalization (OR, 2.66 [95% CI 1.50–4.74]), and non-obese status (underweight status: OR, 3.33 [95% CI 1.05–10.57]; normal weight status: OR, 2.32 [95% CI 1.23–4.38]) in Fig. 3.

## Discussion

### Findings and explanation

We investigated the impact of opioid use on the occurrence of delirium in patients with advanced cancer admitted to APCU. There are several key findings. First, in this large-scale multicenter patient-based registry cohort study that included 2124 eligible patients, opioid use was significantly associated with an increased occurrence of delirium in patients with advanced cancer. Second, the risk of delirium showed a dose-dependent relationship with opioid dose. High-dose opioid users showed higher odds of delirium in patients with advanced cancer. Third, patients with old age, male sex, absence of chemotherapy during hospitalization, and non-obese status showed a significant risk of delirium in stratification analysis.

### Comparison with other studies

Previous studies explored the association between opioid use and delirium in individuals with various surgeries and diseases, including hip fractures, neurological injury, pain, infection, fever, and hypotension ^4,10,11,30^; however, investigations focusing on delirium in patients with advanced cancer are limited. A few studies suggested an association between opioid use and the occurrence of delirium in patients with cancer. However, these studies were limited to case-report studies or small cohort sizes to generalize the results (Table S1) ^6–8,31^. By contrast, our large-scale multicenter patient-based registry cohort, including 2124 eligible patients admitted to the APCU, highlighted the significant association between opioid use and delirium in patients with advanced cancer.

### Possible mechanisms

Opioids are known to exert their effects primarily through the central nervous system by altering neurotransmitter release and neuronal activity^32^. This alternation can lead to neuropsychiatric outcomes, including cognitive impairment and delirium, particularly in patients with advanced cancer^3,4^. Moreover, opioids can disrupt the normal sleep–wake cycle, further exacerbating the risk of delirium^33^. In this cohort study, we found that the association between opioid use and the occurrence of delirium followed a dose-dependent nature. Higher doses of opioids are more likely to induce significant changes in the brain circuits by altering synaptic functions and neural pathways^34^, potentially leading to a higher risk of delirium.

The patients with old age, male sex, absence of chemotherapy during hospitalization, and underweight status showed a significant risk of delirium in stratification analysis. These factors were aligned with identified risk factors for delirium in patients with cancer^9^. Older patients often have decreased physiological reserve and increased sensitivity to opioids, which can predispose them to delirium^35^. Moreover, the significant risk of delirium among male patients could be attributed to a higher incidence of hyperactive forms of delirium in males compared to females^9^. This discrepancy may also suggest potential underdiagnoses of delirium in female patients.

Furthermore, underweight individuals may exhibit different pharmacokinetics and pharmacodynamics, making them more susceptible to delirium^36^. This phenomenon aligns with the concept known as the "Obesity Paradox," where individuals with a higher body mass index (BMI) appear to possess protective factors against postoperative delirium^9,36^. This paradoxical relationship suggests that, similarly, in cancer patients, those with a higher BMI might exhibit a lower risk of delirium, highlighting the complex interplay between body weight and neuropsychiatric outcomes in medical conditions. The absence of chemotherapy during hospitalization could be indicative of a more advanced stage of cancer, where the physiological and psychological burden of the disease itself, coupled with opioid use, could heighten the risk of delirium.

### Policy implications

Our findings not only highlight the need for cautious opioid use in patients with advanced cancer but also emphasize the broader impact of delirium^37^. Delirium poses a significant burden, not only affecting the patients but also placing a significant social burden on their families and healthcare providers^9^. In the context of advanced cancer, delirium adversely impacts the disease course by impairing communication and hindering the participation of patients in care, such as treatment decisions, counseling, and diagnosis^5^. Therefore, policy implications should extend beyond clinical management to include supportive measures for families and caregivers. Healthcare systems should implement policies promoting regular mental status assessments, individualized pain management strategies, and comprehensive support systems for patients with advanced cancer. These measures should be designed to minimize the occurrence of delirium and its associated burdens, thereby improving the overall conditions of patients and families in advanced cancer care contexts.

### Strengths and limitations of the study

This study presents a novel association between opioid use and delirium among patients with advanced cancer by utilizing data from a large-scale, multicenter, patient-based registry cohort. However, several limitations must be acknowledged. First, we collected the information on opioid use relying on the medical records, but they do not necessarily equate to actual consumption of the medication, leading to potential exposure misclassification. Second, while we observed an association, the observational nature of our study precludes a definitive explanation of the causal relationship. It remains unclear whether the association is due to the chronic pain associated with cancer or the opioids themselves. This ambiguity underscores the need for future research to determine the appropriate dosage of opioids that balances analgesic effects and the risk of delirium, as well as to explore the underlying mechanisms. Third, our focus on patients with advanced cancer admitted to the APCU limits the generalizability of our findings to the general patients with cancer. Further studies are needed to assess whether these associations are consistent in patients with less advanced stages of cancer and in different care settings. Fourth, our study is subject to the inherent limitations of a retrospective observational design^38^. The reliance on a patient registry and retrospective chart reviews for evaluating exposures, outcomes, and covariates may introduce bias and affect the generalizability of our findings. To mitigate these limitations, we employed PS matching; however, we acknowledge that it does not fully address the problem^38^. Fifth, the prevalence of delirium observed in our study is lower than in previous studies. However, it is important to note that patients with advanced cancers often exhibit hypoactive delirium, which is more challenging to detect due to its less pronounced symptoms^39^. Thus, the nature of our retrospective chart review study may have resulted in the under-diagnosis of hypoactive delirium.

## Conclusion

In this multicenter patient-based registry cohort study, opioid use was significantly associated with a substantial increase in the risk of delirium in patients with advanced cancer. This association was observed to be dose-dependent, with higher opioid dosages associated with an increased risk of delirium. In addition, we identified various vulnerable groups, including old age, male sex, absence of chemotherapy during hospitalization, and underweight status, for delirium among patients with advanced cancer. These findings highlight the critical need for healthcare providers to carefully prescribe opioids to manage pain in patients with advanced cancer; however, further studies are needed to focus on determining the optimal opioid dosages that minimize the risk of delirium and investigating underlying mechanisms of these associations.

### Supplementary Information

Supplementary Information.

## Data Availability

Data are available on reasonable request. Study protocol, statistical code: available from DKY (email: yonkkang@gmail.com).
