# Supplementary material for: Opioid use and subsequent delirium risk in patients with advanced cancer in palliative care: a multicenter registry study
Source: Sci Rep. 2024 Mar 12;14:6004. doi: 10.1038/s41598-024-56675-1 (PMC10933309; doi:10.1038/s41598-024-56675-1)
Supplement: Supplementary file 1 — Supplementary Information. [file 41598_2024_56675_MOESM1_ESM.docx]

Supplementary Information for

Original article

**Opioid use is associated with an increased risk of delirium in patients with advanced cancer in palliative care: a multicenter, patient-based registry cohort**

Running head: **Opioid use and delirium**

Shin Hye Yoo^1¶^, Jiseung Kang,^2,3¶^ Hyeon Jin Kim,^4,5¶^ Si Won Lee^6,7^, Moonki Hong^6,7^, Eun Hee Jung^8^, Yu Jung Kim ^8^, Dong Keon Yon,^3,4,9*^ Beodeul Kang^10*^

1 Center for Palliative Care and Clinical Ethics, Seoul National University Hospital KR, Seoul, South Korea

2 Department of Anesthesia, Critical Care and Pain Medicine, Massachusetts General Hospital, Boston, MA, USA

3 Division of Sleep Medicine, Harvard Medical School, Boston, MA, USA

4 Center for Digital Health, Medical Science Research Institute, Kyung Hee University College of Medicine, Seoul, South Korea

5 Department of Regulatory Science, Kyung Hee University, Seoul, South Korea

6 Division of Medical Oncology, Department of Internal Medicine, Yonsei Cancer Center, Yonsei University College of Medicine, Seoul, South Korea

7 Palliative Cancer Center, Yonsei Cancer Center, Seoul, South Korea

8 Division of Hematology and Medical Oncology, Department of Internal Medicine, Seoul National University College of Medicine, Seoul National University Bundang Hospital, Seongnam, South Korea

9 Department of Pediatrics, Kyung Hee University Medical Center, Kyung Hee University College of Medicine, Seoul, South Korea

10 Division of Medical Oncology, Department of Internal Medicine, CHA Bundang Medical Center, CHA University School of Medicine, Seongnam, South Korea

^¶^ SHY, JK and HJK are joint first authors.

***Corresponding authors**

**Dong Keon Yon, MD, PhD, FACAAI, FAAAAI**

Department of Pediatrics, Kyung Hee University College of Medicine, 23 Kyungheedae-ro, Dongdaemun-gu, Seoul, 02447, South Korea

Tel: +82-2-6935-2476

Fax: +82-504-478-0201

Email: [yonkkang@gmail.com](mailto:yonkkang@gmail.com)

**Beodeul Kang, MD, PhD**

Division of Medical Oncology, Department of Internal Medicine, CHA Bundang Medical Center, CHA University School of Medicine, 59 Yatap-ro, Bundang-gu, Seongnam 13496, South Korea

Phone: +82-31-780-3438

Fax: +82-31-780-3929

E-mail: wb0707@cha.ac.kr

**Table S1.** Results of a systematic rapid review reporting association between opioid use and risk of delirium in patients with cancer (published, 2000-2023)

| **Author, year** | **Country** | **Samples** | **Subject** | **Data source** | **Results** | **Comparisons with our study** |
| --- | --- | --- | --- | --- | --- | --- |
| Hiratsuka et al., 2023^1^ | Japan | 465 patients | patients with advanced cancer, who received specialized palliative care for cancer pain relief | a multicenter prospective longitudinal study | There was no significant difference in the prevalence of opioid-induced adverse events. | Adverse events were not exclusively related to delirium. Moreover, the cohort size in the referenced study was smaller than in our study (N=2,152 patients with advanced cancer). |
| Mercadante et al., 2022^2^ | Italy | 82 patients | patients with advanced cancer | observational study | There was no significant difference in the prevalence of methadone-induced adverse events at first-line opioid therapy. | Of the opioid medications, only methadone was evaluated, and only adverse events at first-line opioid therapy were investigated. In addition, adverse events were not exclusively related to delirium. Furthermore, the cohort size in the referenced study was smaller than in our study (N=2,152 patients with advanced cancer). |
| Mammana et al., 2021^3^ | Argentina, United States | 62 patients | patients with moderate to severe cancer pain | prospective observational study | There was no significant difference in the prevalence of methadone-induced adverse events at first-line opioid therapy. | Of the opioid medications, only methadone was evaluated, and only adverse events at first-line opioid therapy were investigated. In addition, adverse events were not exclusively related to delirium. Furthermore, the cohort size in the referenced study was smaller than in our study (N=2,152 patients with advanced cancer). |
| Mercadante et al., 2022^4^ | Italy | 113 patients | patients with advanced cancer in two palliative care units | prospective observational study | No patient displayed aberrant behaviours, despite having a moderate-high risk after opioid use. | Adverse events were not exclusively related to delirium. Moreover, the cohort size in the referenced study was smaller than in our study (N=2,152 patients with advanced cancer). |
| Mammana et al., 2021^5^ | Italy | 113 patients | patients with advanced cancer receiving chronic opioid therapy | prospective cross-sectional study | There are barriers to analgesic medication as pain management in patients with advanced cancer. | It is not investigation for opioid use and risk for delirium in patients with advanced cancer. |
| Lim et al., 2018^6^ | Korea, China, United States | 390 patients | Patients with advanced cancer in palliative care | Observational study | Opioid-induced neurotoxicity, including delirium, drowsiness, hallucinations, myoclonus, seizures, and hyperalgesia, were shown in 15% of patients with opioid use. | Opioid-induced neurotoxicity were not exclusively related to delirium. In addition, the cohort size in the referenced study was smaller than in our study (N=2,152 patients with advanced cancer). |
| Fürst et al., 2018^7^ | Sweden | 80 patients | patients with advanced cancer treated in a specialized palliative care unit | retrospective chart review observational study | Methadone treatments increased the risk for sedation and delirium. | Of the opioid medications, only methadone was evaluated, and the cohort size in the referenced study was smaller than in our study (N=2,152 patients with advanced cancer). |
| Hasuo et al., 2016^8^ | Japan | 5 patients | patients with cancer | case reports | The patients with opioid treatments showed delirium. | This study is case reports only involved 5 patients with cancer. |
| Mah et al., 2017^9^ | Canada | 149 patients | patients with advanced cancer | retrospective cohort study | This study compared health-care workers' (HCWs) cancer pain judgments between older patients with advanced cancer with and without a diagnosis of delirium. | It is not investigation for opioid use and risk for delirium in patients with advanced cancer. |
| Reddy et al. 2013^10^ | United States | 190 patients | patients with cancer | observational study | Patients who underwent OR for opioid-induced neurotoxicity. | Opioid-induced neurotoxicity were not exclusively related to delirium. In addition, the cohort size in the referenced study was smaller than in our study (N=2,152 patients with advanced cancer). |
| Moryl et al., 2005^11^ | United States | 20 patients | patients with advanced cancer | clinical trial | Methadone can be effective in the treatment of both refractory pain and what appears to be terminal delirium. | Of the opioid medications, only methadone was evaluated, and the cohort size in the referenced study was smaller than in our study (N=2,152 patients with advanced cancer). |
| Lawlor et al., 2000^12^ | Canada | 113 patients | Patients with advanced cancer in acute palliative care | observational study | Opioid treatments were associated with increased risk of delirium in patients with advanced cancer. | The cohort size in the referenced study was smaller than in our study (N=2,152 patients with advanced cancer). |
| Gagnon et al., 1999^13^ | Canada | 63 patients | Patients with advanced cancer | clinical trial | Oxycodone showed lower risk for delirium in patients with cancer pain compared to other opioids. | The cohort size in the referenced study was smaller than in our study (N=2,152 patients with advanced cancer). |

**References**

1. Hiratsuka Y, Tagami K, Inoue A, et al. Prevalence of opioid-induced adverse events across opioids commonly used for analgesic treatment in Japan: a multicenter prospective longitudinal study. *Support Care Cancer*. Oct 16 2023;31(12):632. doi:10.1007/s00520-023-08099-2

2. Mercadante S, Adile C, Ferrera P, et al. Methadone as First-line Opioid for the Management of Cancer Pain. *Oncologist*. Apr 5 2022;27(4):323-327. doi:10.1093/oncolo/oyab081

3. Mammana G, Bertolino M, Bruera E, et al. First-line methadone for cancer pain: titration time analysis. *Support Care Cancer*. Nov 2021;29(11):6335-6341. doi:10.1007/s00520-021-06211-y

4. Mercadante S, Adile C, Tirelli W, Ferrera P, Penco I, Casuccio A. Aberrant opioid use behaviour in advanced cancer. *BMJ Support Palliat Care*. Mar 2022;12(1):107-113. doi:10.1136/bmjspcare-2020-002606

5. Mercadante S, Adile C, Tirelli W, Ferrera P, Penco I, Casuccio A. Barriers and Adherence to Pain Management in Advanced Cancer Patients. *Pain Pract*. Apr 2021;21(4):388-393. doi:10.1111/papr.12965

6. Lim KH, Nguyen NN, Qian Y, et al. Frequency, Outcomes, and Associated Factors for Opioid-Induced Neurotoxicity in Patients with Advanced Cancer Receiving Opioids in Inpatient Palliative Care. *J Palliat Med*. Dec 2018;21(12):1698-1704. doi:10.1089/jpm.2018.0169

7. Furst P, Lundstrom S, Klepstad P, Runesdotter S, Strang P. Improved Pain Control in Terminally Ill Cancer Patients by Introducing Low-Dose Oral Methadone in Addition to Ongoing Opioid Treatment. *J Palliat Med*. Feb 2018;21(2):177-181. doi:10.1089/jpm.2017.0157

8. Hasuo H, Ishihara T, Kanbara K, Fukunaga M. Myofacial Trigger Points in Advanced Cancer Patients. *Indian J Palliat Care*. Jan-Mar 2016;22(1):80-4. doi:10.4103/0973-1075.173956

9. Mah K, Rodin RA, Chan VWS, Stevens BJ, Zimmermann C, Gagliese L. Health-Care Workers' Judgments About Pain in Older Palliative Care Patients With and Without Delirium. *Am J Hosp Palliat Care*. Dec 2017;34(10):958-965. doi:10.1177/1049909116672641

10. Reddy A, Yennurajalingam S, de la Cruz M, et al. Factors associated with survival after opioid rotation in cancer patients presenting to an outpatient supportive care center. *J Pain Symptom Manage*. Jul 2014;48(1):92-8. doi:10.1016/j.jpainsymman.2013.08.010

11. Moryl N, Kogan M, Comfort C, Obbens E. Methadone in the treatment of pain and terminal delirum in advanced cancer patients. *Palliat Support Care*. Dec 2005;3(4):311-7. doi:10.1017/s1478951505050479

12. Lawlor PG, Gagnon B, Mancini IL, et al. Occurrence, causes, and outcome of delirium in patients with advanced cancer: a prospective study. *Arch Intern Med*. Mar 27 2000;160(6):786-94. doi:10.1001/archinte.160.6.786

13. Gagnon B, Bielech M, Watanabe S, Walker P, Hanson J, Bruera E. The use of intermittent subcutaneous injections of oxycodone for opioid rotation in patients with cancer pain. *Support Care Cancer*. Jul 1999;7(4):265-70. doi:10.1007/s005200050259
